# Higher Physical Activity Levels May Help Buffer the Negative Psychological Consequences of Coronavirus Disease 2019 Pandemic

**DOI:** 10.3389/fpsyg.2021.672811

**Published:** 2021-04-22

**Authors:** Raul Antunes, Ricardo Rebelo-Gonçalves, Nuno Amaro, Rogério Salvador, Rui Matos, Pedro Morouço, Roberta Frontini

**Affiliations:** ^1^CIEQV – Life Quality Research Centre, Polytechnic of Leiria, Leiria, Portugal; ^2^ESECS, Polytechnic of Leiria, Leiria, Portugal; ^3^Center for Innovative Care and Health Technology (ciTechCare), Polytechnic of Leiria, Leiria, Portugal; ^4^Research Unit for Sport and Physical Activity (CIDAF – uid/dtp/04213/2020), University of Coimbra, Coimbra, Portugal

**Keywords:** coronavirus (2019-nCoV), motivation, public health, physical activity, exercise, anxiety

## Abstract

This study explored the associations between physical activity (PA) anxiety levels, and the perception of satisfaction of basic psychological needs (BPN), during Coronavirus Disease 2019 (COVID-19) lockdown. Thus, 1,404 participants (977 women, 426 men, and one respondent preferred not to answer) ranging from 18 to 89 years old (36.4 ± 11.7 year-old) completed a questionnaire in the period between 1st and 15th April 2021. The survey included sociodemographic data and the following validated instruments: the International Physical Activity Questionnaire (IPAQ), the Basic Need General Satisfaction Scale and the State-Trait Anxiety Inventory. The Kruskal-Wallis test was performed to examine variation in anxiety levels and BPN satisfaction according to PA category (low, moderate, and high). Spearman’s Rho correlations coefficients were used to determine the association between anxiety levels and psychological needs. Individuals presenting a higher level of PA revealed lower levels of anxiety-state (*H* = 20.14; *p* < 0.01). Differences between elements from different levels of PA were found for the autonomy (*H* = 23.52; *p* < 0.001), competence (*H* = 18.89; *p* < 0.001), and relatedness (*H* = 24.42; *p* < 0.001) psychological needs, suggesting that those who feel their BPN as more satisfied have higher levels of PA. The study found statistically significant correlations between anxiety-state and the satisfaction of the needs for autonomy (*p* = 0.01; *r* = −0.46), competence (*p* = 0.01; *r* = −0.40), and relatedness (*p* = 0.01; *r* = −0.21). These findings support the importance that PA has in the anxiety levels during social isolation, emphasizing the importance of multidisciplinary teams in an individual-based approach.

## Introduction

On the 30th January 2020, the WHO (World Health Organization, 2020a) declared the new Coronavirus Disease 2019 (COVID-19) as public health emergency of international concern and later as a pandemic, as the SARS-CoV-2 virus spread worldwide among humans at an exponential rate. Several countries adopted procedures to prevent the spread of the virus such as the restriction of the people’s free movement and circulation (World Health Organization, 2020b). Among these procedures, quarantine (i.e., the control of activities or separation of non-sick people to monitor their symptoms and detect earlier cases) and social isolation (i.e., the separation of infected people from the remaining population to prevent the spread of the infection) were applied as part of wider actions (World Health Organization, 2020c).

The state of emergency was declared in Portugal on March 18th by the President (Decree of the President of the Republic no. 14-A/2020). Thereafter, the Portuguese Government dictated a set of mandatory rules promoting social isolation. For example, gyms, health clubs, and sports centers were closed and all forms of collective exercise were also prohibited. Nevertheless, it was possible, during this time (during the first lockdown), to make short trips with the purpose to practice physical activity (PA).

These social isolation measures, as well as quarantine measures, may have a positive effect in protecting peoples’ physical health. However, while preventing and mitigating the virus transmission, the application of these restraint initiatives can have a long-lasting and wide-ranging negative psychosocial impact. Indeed, they may induce the separation of significant relatives, and the perception of isolation, the loss of freedom and the demanding restructure of a new lifestyle (Hawryluck et al., 2004; Jeong et al., 2016; Antunes et al., 2020; Brooks et al., 2020).

Literature acknowledged several psychological effects of social isolation such as high levels of anxiety (Antunes et al., 2020), stress, or fear that can persist beyond that period (Jeong et al., 2016; Brooks et al., 2020). Moreover, the implementation of these actions may have a greater physical and mental effect on peoples’ who are used to practice regular physical activities (Hammami et al., 2020), regardless it is home-based or outdoor-based activities. Thus, it is crucial to explore the psychological effects of social isolation particularly considering that positive social interactions may be considered as basic human needs causing social cravings similar to food cravings (Tomova et al., 2020). The theory of basic psychological needs (BPN) argues that a subject’s motivation is directly related to factors of social involvement because the influence of these factors is mediated by the satisfaction of all three BPNs (Deci and Ryan, 2008). The three BPN, comprehend autonomy (i.e., the subject’s ability to regulate his or her own actions), competence (i.e., the subject’s efficiency in interaction with the environment), and relatedness (i.e., the subject’s ability to search for and develop connections and interpersonal relationships; Deci and Ryan, 2000).

Considering the possible negative consequences of this period, particularly by increasing the levels of stress and anxiety, the WHO emitted a set of deliberations (World Health Organization, 2020d). These aim to minimize the negative effects, highlighting the need to maintain family routines and to seek a healthy lifestyle through regular PA, healthy eating, and regular sleep routines. The practice of PA may have a fundamental role, as changes in daily routines may lead to an increase in sedentary behaviors and anxiety levels (Chen et al., 2020). Frequent PA not only has unquestionable benefits for physical health (Bentlage et al., 2020), but it is also associated with a decrease in anxiety levels (Stubbs et al., 2017; Gammon and Hunt, 2018). Although this pandemic and its consequence are still recent, the available studies already demonstrated a trend for the impact of social isolation on people’s physical and mental health (Lesser and Nienhuis, 2020; Roma et al., 2020; Hawes et al., 2021). Still, the consequences on BPN are not fully known.

The aim of this study was to examine if subjects with different levels of PA [determined by resorting to the International Physical Activity Questionnaire (IPAQ) physical activity categories], reported different anxiety levels and different perceptions of satisfaction of BPN, during the isolation period due to COVID-19. Thus, the association between PA, anxiety, and psychological needs were explored, using self-reported measures in a non-representative sample of Portuguese volunteers during the first COVID-19 lockdown. It was hypothesized that individuals who reported higher levels of physical activity would report lower levels of anxiety and higher perceptions of satisfaction of three BPN. It also expected that physical activity was associated with BPN and anxiety. It was also hypothesized that gender moderated the relationship between anxiety state and anxiety trait.

## Materials and Methods

### Study Design and Procedures

This is a cross-sectional design conducted in the period between April 1st and 15th, 2020, during which a state of emergency was decreed in Portugal. Social media (Facebook and Instagram) and regional newspapers (both in digital format and in paper) were primary vehicles to advertise and recruit volunteers to take part in this study. Participants completed a questionnaire through google forms, which included four domains: sociodemographic data, PA levels, anxiety, and BPN satisfaction. There was no compensation or reimbursement involved for study participants.

Procedures followed standards for research in sports medicine and were performed according to the Declaration of Helsinki. Participants were fully informed about the nature of the study and the procedures involving data recording. Participants were voluntary, could withdraw from the study at any time and provided informed consent before the questionnaire’s completion. Anonymity was guaranteed.

### Participants

Subjects were recruited by convenience sample method and were only eligible if they were aged over 18 years old and had Portuguese nationality. A total of 1,404 respondents participated (36.4 ± 11.7 years of age), ranging from 18 to 89 years old. The sample comprised 977 women (69.6%) aged 35.7 ± 11.6 years, 426 men (30.3%) aged 38.1 ± 11.6 years, and one respondent preferred not to answer (35.0 years old; 0.1%). This survey, involving community adults, intended to embrace several domains of an individual’s behavior and feelings toward the confinement period of COVID-19. The survey sociodemographic questions were developed and reviewed by four experts in the areas of Exercise, Sports, and Psychology. The remain domains assessment comprised validated instruments.

### Variables

#### Sociodemographic Characterization

Participants were required to self-report age, gender, marital status, living status during the COVID-19 and academic level.

#### Anxiety

The Portuguese version (Silva, 2003) of the State-Trait Anxiety Inventory (STAI-state, STAI-trait; Spielberger et al., 1983) was used. This questionnaire is composed of two blocks (Form 1 and Form 2) of 20 statements, evaluated in a four-point Likert scale. Form 1 – STAI-State, evaluates transient or temporary anxiety, i.e., the anxiety the person is feeling at the present moment. The form 2 – STAI-Trait assesses dispositional or general anxiety. The score is generated by the sum of the 20 items for each scale. Higher levels correspond to higher anxiety levels. Internal consistency in this study proved to be good (state *α* = 0.93; trait *α* = 0.93).

#### Basic Psychological Needs

The Portuguese validated version of the Basic Need General Satisfaction Scale (BNSG-S; Gagné, 2003) was used (Sousa et al., 2012). This questionnaire comprises 21 items, to which the subjects respond using a seven-point “Likert” scale ranging from one (“totally disagree”) to seven (“totally agree”). The items are grouped into three factors (autonomy, competence, and relatedness). Internal consistency for the subscales, in this study, ranged from acceptable to good (autonomy *α* = 0.65; competence *α* = 0.78; and relatedness *α* = 0.66).

#### Physical Activity

The Portuguese validated version of The International Physical Activity Questionnaire (IPAQ-short form) was used to assess PA (Craig et al., 2003). This questionnaire is formed of four questions related to specific types of PA, e.g., walking and moderate and vigorous activities, in terms of the frequency and duration of each specific type of activity, and the time spent seated per day in a week. The data obtained by the IPAQ is converted into MET-min/week (metabolic equivalent) through the calculation of the marked minutes per week in each category of activities by their specific metabolic equivalent (Walking = 3.3 METs; Moderate PA = 4.0 METs; Vigorous PA = 8.0 METs). The physical activity level of each individual is ranked according to the IPAQ’s recommendations, which present the following physical activity categories:

Category 1 (Low) – The lowest physical activity level, which corresponds to individuals who do not fulfill the criteria for categories 2 and 3, who are considered to be inactive; Category 2 (Moderate) – Individuals who meet one of the following criteria: (a) 3 or more days of vigorous physical activity for at least 20 min a day; (b) 5 or more days of any combination of walking, or moderate or vigorous physical activity, which reaches a total minimum of physical activity of at least 600 MET-min/week; Category 3 (High) – Individuals who meet one of the following criteria: (a) vigorous activity for at least 5 days, reaching a total minimum of physical activity of 1,500 MET-min/week; (b) 7 or more days of any combination of walking, or moderate or vigorous activities, which reach a total minimum of physical activity of at least 3,000 MET-min/week.

### Data Analysis

Counts (and proportions), means, SD 95% CI (95% CI), and medians (interquartile range, IQR) were computed to describe both categorical and continuous variables for the total sample. Normality was checked using the Shapiro-Wilk test and by visual inspection of normality plots. The assumptions of normal distribution were violated for all variables.

Participants were grouped according to PA level (low, moderate, and high). The Kruskal-Wallis test was used to compare groups for anxiety levels and the perception of satisfaction of BPN. When the results for the dependent variables were significant, appropriated procedures for multiple comparisons between groups were executed.

The reported estimate of effect size measures following the recommendations for non-parametric tests (Tomczak and Tomczak, 2014). Those estimates assumed values ranging from 0 to 1; multiplied by 100 indicates the percentage of variance in the dependent variable explained by the independent variable. According to Hopkins et al. (2009) the magnitude of the correlation coefficient was considered as trivial (*r* < 0.1), small (0.1 < *r* < 0.3), moderate (0.3 < *r* < 0.5), large (0.5 < *r* < 0.7), very large (0.7 < *r* < 0.9), and nearly perfect (*r* > 0.9). Significance was set at 5%.

Spearman’s Rho correlations coefficients were used to determine the association between the selected variables. Data analysis was performed using the IBM Statistical Package for Social Science software for Windows (v.26.0, IBM Corp.; Armonk, NY, United States).

The moderation was performed using Model 1 of PROCESS (Hayes, 2013), an SPSS macro for path analysis-based moderation. PA was used as the independent variable and anxiety-state as the dependent variable. Gender was the moderator. A bootstrapping procedure was used (with 10.000 resamples). Significance was set at the 0.05 level.

## Results

The sample characteristics are presented in Table 1. Nearly half the participants (49.6%) were classified as moderate in the IPAQ categories. Mean values for anxiety-state (45.1 ± 11.2), anxiety-trait (37.9 ± 10.3) and the BPN factors were reported for the total sample.

**Table 1:** Summary of descriptive statistics (*n* = 1,404).

	*n* (%)	Mean		Median (IQR)
		mean ± SD	(CI 95%)	
Age (years)		36.4 ± 11.7	(35.8–37.0)	37.0 (18.0)
Marital status				
Single	620 (44.2)			
Married	642 (45.7)			
Separated	16 (1.1)			
Divorced	108 (7.7)			
Widower	9 (0.6)			
Other	9 (0.6)			
Living status – COVID 19				
In social isolation at home, not working and alone	40 (2.8)			
In social isolation at home, not working, with other people	472 (33.6)			
Working out in full-time	136 (9.7)			
Working out in part-time	104 (7.4)			
Teleworking at home, alone	72 (5.1)			
Teleworking at home, with other people	575 (41)			
Home quarantine	5 (0.4)			
Academic level				
Elementary	50 (3.6)			
Secondary	263 (18.7)			
Professional	107 (7.6)			
Superior	984 (70.1)			
IPAQ categories				
Low	447 (31.8)			
Moderate	697 (49.6)			
High	260 (18.5)			
Total energy expenditure (METS)		1843 ± 2,155	(1730–1956)	1,206 (1942)
Anxiety-state		45.1 ± 11.2	(44.5–45.7)	44.0 (15.0)
Anxiety-trait		37.9 ± 10.3	(37.4–38.4)	36.0 (13.0)
Autonomy		4.43 ± 0.67	(4.40–4.47)	4.43 (0.86)
Competence		5.01 ± 0.88	(4.97–5.06)	5.00 (1.13)
Relatedness		4.95 ± 0.58	(4.92–4.98)	5.00 (0.88)

SD, standard deviation; CI 95%, confidence interval 95%; IQR, interquartile range.

Variation associated with PA level showed significant differences among groups in both anxiety-state and anxiety-trait, and in all BPN (Table 2). The results seem to point to two interesting trends from the low PA level category to the high PA level category: a decreasing trend in anxiety-state and anxiety-trait; an increasing trend in autonomy, competence, and relatedness.

**Table 2:** Comparison between the International Physical Activity Questionnaire (IPAQ) categories, anxiety levels and basic psychological needs (BPN; *n* = 1,404).

	IPAQ category 1 Low (*n* = 447)		IPAQ category 2 Moderate (*n* = 697)		IPAQ category 3 High (*n* = 260)		Kruskal-Wallis test value	*Post hoc*
	mean ± SD	median	mean ± SD	median	mean ± SD	median		
Anxiety-state	46.94 ± 11.51	46.00	44.79 ± 11.04	44.00	42.68 ± 10.40	42.00	20.14^**^	2 > 3 1 > 3 1 > 2
Anxiety-trait	39.70 ± 10.71	39.00	37.42 ± 9.88	36.00	36.01 ± 9.98	34.00	23.11^**^	1 > 2 1 > 3 2 > 3
Autonomy	4.30 ± 0.70	4.28	4.46 ± 0.65	4.42	4.56 ± 0.63	4.57	23.52^**^	2 > 1 3 > 1 2 > 3
Competence	4.86 ± 0.87	4.83	5.05 ± 0.84	5.00	5.16 ± 0.91	5.16	19.89^**^	2 > 1 3 > 1
Relatedness	4.79 ± 0.57	4.87	4.99 ± 0.57	5.00	5.03 ± 0.57	5.00	24.42^**^	3 > 1 2 > 1

** *p* < 0.001.

Results for the association between anxiety levels and BPN are summarized in Table 3. Significant negative associations were noted between both anxiety levels and three basic psychological needs: autonomy, competence, and relatedness.

**Table 3:** Spearman’s *ρ* correlations between anxiety levels and self-reported BPN (*n* = 1,404).

	Anxiety-state	Anxiety-trait	Autonomy	Competence	Relatedness
Anxiety-trait	0.670^**^	-	-	-	-
Autonomy	−0.468^**^	−0.494^**^	-	-	-
Competence	−0.401^**^	−0.613^**^	0.512^**^	-	-
Relatedness	−0.214^**^	−0.323^**^	0.349^**^	0.462^**^	-
Total energy expenditure	−0.148^**^	−0.140^**^	0.135^**^	0.119^**^	0.119^**^

** *p* < 0.001.

### Moderation Analyses

**Figure 1:**
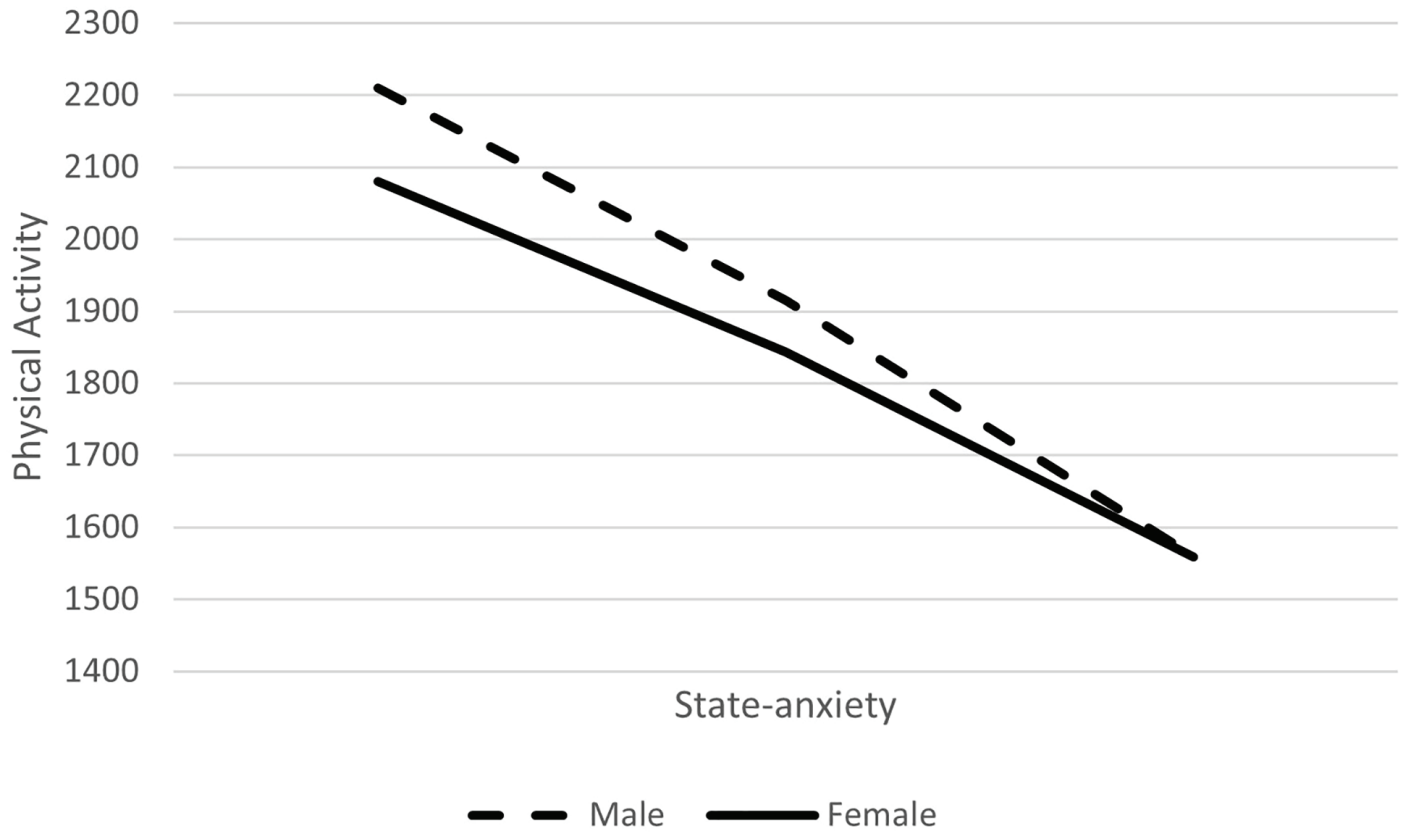
The possible moderation role of gender in the relationship between physical activity (PA; MET-min/week) and state-anxiety.

To test the moderating effect of gender on the associations between PA and state-anxiety path analysis-based moderation was performed. Next, using ModGraph (Jose, 2013) this significant interaction was plotted (see Figure 1). Results showed that higher levels of PA were related to lower levels of state-anxiety, but this association occurred in both males and females.

## Discussion

This study explored the associations between PA, assessed by the IPAQ questionnaire, anxiety levels, and perception of satisfaction of basic psychological needs, during COVID-19 lockdown. Our main findings suggest that people with higher levels of PA present higher values of satisfaction of autonomy, competence, and relatedness, and lower levels of anxiety, both when we analyze PA category-related variation, and when testing the moderating effect of gender on the associations between PA and state-anxiety.

Regarding the anxiety-state, we verified significant differences between the three levels of PA (*H* = 20.14; *p* =< 0.01; $\eta_{H}^{2}$ = 0.013). Individuals presenting a higher level of PA showed lower levels of anxiety, which meets the conclusions of several studies that stated that PA is crucial to reduce anxiety levels (Stubbs et al., 2017; Lesser and Nienhuis, 2020; Meyer et al., 2020; Pons et al., 2020). This is particularly important considering the current pandemic and all the changes associated with it, along with the impact that this period has by being a source of anxiety (Chen et al., 2020; Lai et al., 2020). Some studies showed that one of the direct consequences of COVID-19 is the rise in anxiety levels, which as been found in Portugal (Frontini et al., 2021), and in other countries such as China (Hou et al., 2020; Pieh et al., 2020; Wang et al., 2020), Austria (Xiang et al., 2020) or Republic of Ireland (Hyland et al., 2020).

Concerning the BPN, with higher levels of PA were those who felt their BPN as more satisfied; significant differences were observed for autonomy (*H* = 23.52; *p* =< 0.001), competence (*H* = 18.89; *p* =< 0.001), and relatedness (*H* = 24.42; *p* =< 0.001). These results are in line with previous investigations, stating that the higher the satisfaction’s perception of their basic psychologic needs the more self-determined (more autonomous) is the behavior (Deci and Ryan, 2000) which in turn can contribute to higher levels of PA (Almagro et al., 2011; Sicilia et al., 2014). In fact, previous research showed a positive relationship between meeting these three BPN and greater intrinsic motivation (Franco et al., 2017), which is recognized to be fundamental for greater PA practice (Ryan and Deci, 2000). According to the theoretical model and considering an isolation period such as the one we are living in, the satisfaction of BPN can be influenced by the alteration of behaviors due to changes in relationships or interaction context (La Guardia and Patrick, 2008). The same applies to a regular practice of PA and exercise (Fraguela-vale et al., 2020).

By promoting the satisfaction of BPN, the person may feel more self-determined and self-sufficient for the practice of PA, promoting higher levels of their practice which, in turn, will have not only positive repercussions in their physical health but also a positive impact on their mental health, namely in anxiety levels. Thus, with these psychological resources available, the individual may feel more confident to engage in PA in social isolation periods.

In this study, people with higher levels of satisfaction of BPN were engaged in higher levels of PA, which has several implications. Regarding relatedness, exercise physiologists dealing with people in social isolation (e.g., due to a disease, a pandemic or even due to a new COVID-19 wave) may integrate relatedness in addition to PA. Nowadays, with the massive use of social networks and technological devices such as the smartphone, computer, or tablets, this contact can be established even if the person is in social isolation. In fact, online training is considered number one in fitness trends for 2021 (Thompson, 2021). To promote autonomy, exercise physiologists must consider the importance of particular exercises for the physical and mental health of the practitioner, meeting their personal goals and interests (Ntoumains and Mallett, 2014). The individual should be encouraged to participate in the decision-making construction of the exercise-plan and the exercise physiologist should understand the individual’s perspective before offering suggestions. A critical procedure that has been recognized as important for engagement and for the establishment of autonomy in sports and exercise psychology (Mageau and Vallerand, 2003). Since people are in social isolation the exercise physiologist may bear in mind that the individual must use personal material that can be found at home which, in turn, will meet the satisfaction of the need for autonomy. This autonomous work can later be continued at a gym after the end of confinement. Considering the need for competence, it may be important to prescribe training programs promoting individuals’ skills, providing clear indications on success criteria, empowering the person to assess his/her progression.

It is imperative that exercise physiologists are able to provide the satisfaction of BPN, in this context of social confinement. That will lead to internalizing behaviors, to more self-determined levels, in order to create conditions for the subjects to maintain this type of regulation of motivation that is usually associated with greater maintenance of behavior, commitment, persistence, and fun in the activity performed (Deci and Ryan, 2000).

The media and the Public Health Portugal have been advocating PA as crucial to individuals physical and mental health. It is thus important that future studies on COVID-19 or on social isolation and/or quarantine should try to understand if there were visible changes in PA practice and possible consequence on anxiety levels and/or mental health in general. It will be interesting to understand if someone starts practicing physical activities during the isolation period and if this habit is kept afterward. Looking for related differences could also be interesting. Future studies should consider the potential effect that PA could have on anxiety, which might be enhanced by an environment that promotes the satisfaction of BPN.

Regarding the analysis correlations, there were statistically significant correlations between state-anxiety and the satisfaction of the needs for autonomy (*p* = 0.01; *r* = −0.46), competence (*p* = 0.01; *r* = −0.40), and relatedness (*p* = 0.01; *r* = −0.21), which in line with literature that refers that BPN negatively predicts anxiety (Brunet and Sabiston, 2009; Quested et al., 2011). Further research is needed to better understand the relationship between these variables. In fact, it is possible that the correlation between these variables is low because the relationship between them is not direct (Hayes, 2013). Rather, other variables may, for instance, mediate the relationship between needs for autonomy, competence, and relatedness and anxiety. Further studies, namely path analysis, are needed. To further deepen this relationship, we tested the possible moderating role of gender in this relationship and, through a moderation analysis, we probed our knowledge regarding the relationship between PA and state anxiety. Our results showed that the relationship between the two variables does exist, but it is independent of gender.

Some limitations should be noted: the cross-sectional design of the study, which does not allow causal inferences to be made and the convenience sample enrolled with the recruitment being online and which does not allow the generalization of results Thus, results should be interpreted with caution. However, the present study presents several strengths. When studying individual concepts such as anxiety or satisfaction with psychological needs, self-reports are vital and the ideal strategy. Moreover, the sample size is notable, especially considering it was difficult to conduct any objective measurements during the COVID-19 pandemic.

## Conclusion

These findings may help develop ways to support people psychologically and socially during this outbreak, in case of future pandemics and/or future waves or purely in case of social isolation and/or quarantine. Understanding how to help fight the psychological consequences of social isolation and quarantine is important to advise policymakers and healthcare practitioners. It would be vital to enhance the practice of PA in contexts of social isolation and/or pandemic crises, in association with a climate that promotes the satisfaction of the BPN, which could buffer and reduce anxiety levels.

Our findings support previous studies regarding the importance that PA may have in the anxiety level. Thus, in the case of future intervention programs aiming to help individuals dealing with social isolation, PA may be an important component, along with a motivational and psychological work regarding the satisfaction of BPN. In fact, our results underline the importance that the promotion of BPN may have in reducing anxiety.

## Data Availability Statement

The raw data supporting the conclusions of this article will be made available by the authors, without undue reservation.

## Ethics Statement

Ethical review and approval was not required for the study on human participants in accordance with the local legislation and institutional requirements. The patients/participants provided their written informed consent to participate in this study.

## Author Contributions

RA was the leader of the research group that conducted the study. RF, RA, RR-G, NA, RS, RM, and PM contributed to the conception and design of the study. RF, RA, and RR-G organized the database. RF and RA performed the statistical analysis and wrote the first draft of the manuscript. NA, RS, RM, PM, and RR-G reviewed and edited the first draft. All authors contributed to the article and approved the submitted version.

### Conflict of Interest

The authors declare that the research was conducted in the absence of any commercial or financial relationships that could be construed as a potential conflict of interest.
